# Urban canine leishmaniasis in an Amazonian municipality: a cross-sectional study of prevalence, distribution and phlebotomine fauna during the dry season, Brazil, 2023

**DOI:** 10.1590/S2237-96222025v34e20240130.en

**Published:** 2025-06-13

**Authors:** Adriana Sousa Tapajós, Luciana Pinto Oliveira, Andréa Helena Martins Amaral, Clístenes Pamplona Catete, Walter Souza Santos, Lourdes Maria Garcez

**Affiliations:** 1 Secretaria de Estado de Saúde Pública do Pará, Diretoria de Endemias, Belém, PA, Brazil; 2 Instituto Evandro Chagas, Laboratório de Epidemiologia das Leishmanioses, Ananindeua, PA, Brazil; 3 Universidade do Estado do Pará, Centro de Ciências Biológicas e da Saúde, Belém, PA, Brazil

**Keywords:** Canine Leishmaniasis, Polymerase Chain Reaction, Geographic Risk Location, Psychodidae, Cross-Sectional Studies, Leishmaniosis Canina, Reacción en Cadena de la Polimerasa, Localización Geográfica de Riesgo, Psychodidae, Estudios Transversales

## Abstract

**Objective:**

To describe the prevalence and distribution of canine leishmaniasis and the phlebotomine fauna across the five urban neighborhoods of the Cachoeira do Piriá Amazonian municipality, Pará state, Brazil, during the dry season.

**Methods:**

Dogs were sampled for five hours in each neighborhood, with blood and conjunctival swab specimens (from June 13 to 15, 2023). Samples were tested using polymerase chain reaction targeting the *heat shock protein 70-234* gene. Risk areas (kernel) and phlebotomine fauna were investigated (from 1 to 7/10/2023).

**Results:**

A total of 93/864 (11%) animals were included. The prevalence of canine leishmaniasis was 65% (60/93). Positivity rates varied among neighborhoods (p-value 0.001), with major clusters in the Northwest and Southeast urban areas, encompassing three neighborhoods. *Lutzomyia antunesi* (2) and *Lutzomyia evandroi* (11) were found in four neighborhoods.

**Conclusion:**

High prevalence of canine leishmaniasis and a potential phlebotomine vector (*Lutzomyia antunesi*) were identified during the Amazonian summer in the urban area of Cachoeira do Piriá, where three neighborhoods were prioritized for surveillance.

## Resumen

**Objetivo:**

Describir la prevalencia y distribución de la leishmaniosis canina y la fauna de flebotomíneos en los cinco barrios urbanos del municipio amazónico de Cachoeira do Piriá, estado de Pará, Brasil, durante la estación seca.

**Métodos:**

Se realizaron muestreos de perros durante cinco horas en cada barrio, recolectando muestras de sangre y hisopados conjuntivales (del 13 al 15/06/2023). Las muestras fueron analizadas mediante reacción en cadena de la polimerasa dirigida al gen heat shock protein 70-234. Se investigaron las áreas de riesgo (kernel) y la fauna de flebotomíneos (del 1 al 7/10/2023).

**Resultados:**

Se incluyeron 93 de 864 animales (11%). La prevalencia de leishmaniosis canina fue del 65% (60/93). Las tasas de positividad variaron entre los barrios (p-valor 0,001), identificándose principales conglomerados al Noroeste y Sureste de la zona urbana, que abarcaron tres barrios. Se encontraron *Lutzomyia antunesi* (2) y *Lutzomyia evandroi* (11) en cuatro barrios.

**Conclusión:**

Se describió una alta prevalencia de leishmaniosis canina y un vector potencial de flebotomíneos (*Lutzomyia antunesi*) durante el verano amazónico en la zona urbana de Cachoeira do Piriá, donde tres barrios son prioritarios para la vigilancia.

**Ethical aspects**

This research respected ethical principles, having obtained the following approval data. :

Research Ethics Committee: Instituto Evandro Chagas

Opinion number: 04/2023

Approval date: 14/4/2023

Certificate of Submission for Ethical Appraisal: 04/2023

Informed Consent Form: This research did not involve human participants. Written consent for sample collection was obtained from all dog owners.

## Introduction

The domestic dog serves as a reservoir host for *Leishmania infantum*, the etiological agent of visceral leishmaniasis (1), and has also been implicated as a reservoir for species that cause tegumentary leishmaniasis (2-3).

The vectorial capacity of phlebotomine sand flies depends on species density, their ability to colonize modified environments, and their competence in feeding on human blood (4). The species *Lutzomyia antunesi*, which has been found naturally infected with *Leishmania naiffi* in Rondônia state (5) and Leishmania *lindenbergi* in Pará (6), and is frequently reported in the northeast region of Pará state (7-9), is considered a potential vector of Leishmania.

This study describes the canine leishmaniasis prevalence and distribution and the phlebotomine fauna in the urban area of Cachoeira do Piriá, a municipality in northeastern Pará, during the Amazonian summer.

## Methods

### Design

This was a descriptive cross-sectional study conducted in the urban area of the Cachoeira do Piriá municipality, located in northeastern Pará state, Amazon Region, Brazil.

### Background

Northeastern Pará represents the oldest colonization frontier in the state (10), where Cachoeira do Piriá frequently undergoes landscape changes, including those caused by gold mining in urban areas. From 2013 to 2022, 324 cases of tegumentary leishmaniasis were reported in the municipality, which has a population of 19,630 inhabitants, an area of 2,419 km^2^ (11) and only five urban neighborhoods. Local surveillance teams do not monitor canine leishmaniasis.

### Sampling of urban dogs

Blood samples (2-3mL) and conjunctival swabs were collected from dogs at a fixed 5-hour period per sampling unit (neighborhood), through active search guided by the 2022 canine urban census (864 dogs). Blood samples in ethylenediaminetetraacetic acid and preservative-free conjunctival swab were stored, transported (4 ^o^ -8^o^ C/3h) and preserved (-20^o^ C) until deoxyribonucleic acid (DNA) extraction.

### Variables and statistics

Variables: a) prevalence, distribution and density of canine leishmaniasis cases; b) frequency and distribution of phlebotomine species; c) sensitivity of the polymerase chain reaction (PCR) based on the targeting the *heat shock protein 70-234* (2), with DNA extracted from blood and conjunctival swab for canine leishmaniasis diagnosis.

Sensitivity of PCR for DNA extracted from blood and conjunctival swab (Fisher’s test) and the proportions of positive dogs were compared among the five urban neighborhoods (chi-square of independence) of Cachoeira do Piriá (α: 0.05; BioEstat, version 5.0).

### Diagnosis of canine leishmaniasis

After DNA extraction (Wizard Genomic DNA Purification - Promega), with a final volume of 50mL, the PCR conditions were as follows: Mix 50mL (Taq *DNA* polymerase 0.03U/µL; MgCl2 1.5mM; Invitrogen KCI buffer; dNTPs 0.25mM; primers F- 5’ GGA CGA GAT CGA GCG CAT GGT 3’ and R- 5’ TCC TTC GAC GCC TCC TGG TTG 3’, 0.2pM each; DNA 3.0µL); denaturation, annealing and extension (1x 94° C/5’; 32x 94° C/0.5’, 61º C/1’ and 72° C/1’); and final extension (72° C/10’). Amplification of the target (*heat shock protein 70*-234), visualized on agarose gel, confirmed the presence of *Leishmania* DNA in the sample (2).

### Collection and identification of sand flies

From October 1 to 7, 2023, 14 Center for Disease Control (CDC) light traps were installed, in peri- and intra-household areas (6pm-6am), over three consecutive nights, in the five neighborhoods of Cachoeira do Piriá, at seven capture points: Vitória (1); Piçarreira (2); Centro (1); São José (2) and Cachoeira Velho (1). The total entomological capture effort was 180 hours. Phlebotomine sand flies were preserved (in 70% ethanol), clarified, diaphanized, mounted between the slide and coverslip using Berlese fluid and morphologically identified (12-14).

## Results

A total of 93/846 (11%) urban dogs, both male and female, aged 6 months or older, symptomatic or asymptomatic for leishmaniasis, were included.

**Figure 1:**
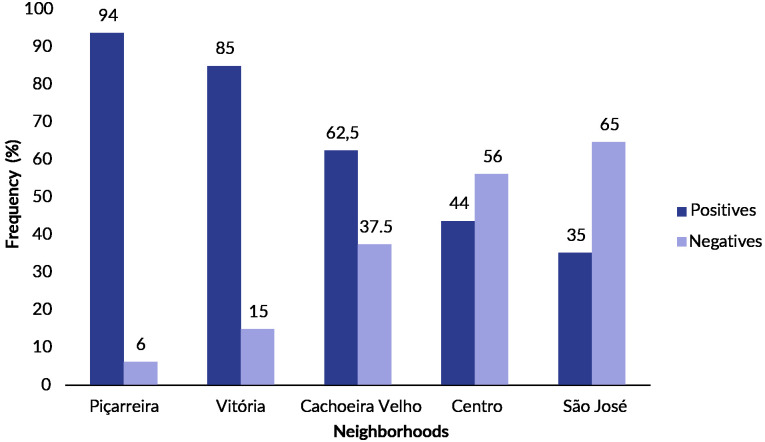
Rates of positive and negative results of polymerase chain reaction for canine leishmaniasis, based on the *heat shock protein 70*-234 gene sequence, across five urban neighborhoods. Cachoeira do Piriá, 2023 (p-value 0.001)

The prevalence of canine leishmaniasis was 65% (60/93) in the urban environment, according to PCR results (Table 1). Positivity rates by neighborhood (Figure 1) ranged from 35% to 94% (ꭓ^2^: 19.036; 4DF; p-value 0.001).

**Table 1:** Polymerase chain reaction (PCR) based on the *heat shock protein 70*-234 gene sequence, for the diagnosis of canine leishmaniasis and the phlebotomine fauna during the dry season across five urban neighborhoods. Cachoeira do Piriá, 2023

Urban neighborhoods	PCR for canine leishmaniasis		Total n (%)	Phlebotomine species in peridomicile	Sex		Total n (%)
	Positives	Negatives			Male	Female	
Picarreira	15	1	16 (17.0)	-	0	0	0
Victória	17	3	20 (22.0)	*Evandromyia evandroi*	1	3	4 (31.0)
Cachoeira Velho	15	9	24 (26.0)	*Evandromyia evandroi*	1	5	6 (46.0)
	7	9	16 (17.0)	*Nyssomyia antunesi*	0	1	1 (8.0)
São José	6	11	17 (18.0)	*Nyssomyia antunesi*	0	1	1 (8.0)
				*Evandromyia evandroi*	1	0	1 (8.0)
Total	60	33	93 (100)		3	10	13 (100)

There was a significant difference in test sensitivity depending on the sample (p-value 0.001): DNA extracted from blood (53/93; 57%); or conjunctival swab (32/93; 34%).

Phlebotomine sand flies were found in the peri-domestic environment of four neighborhoods, with the exception of Piçarreira. Two species were represented in the sample (Table 1).

**Figure 2:**
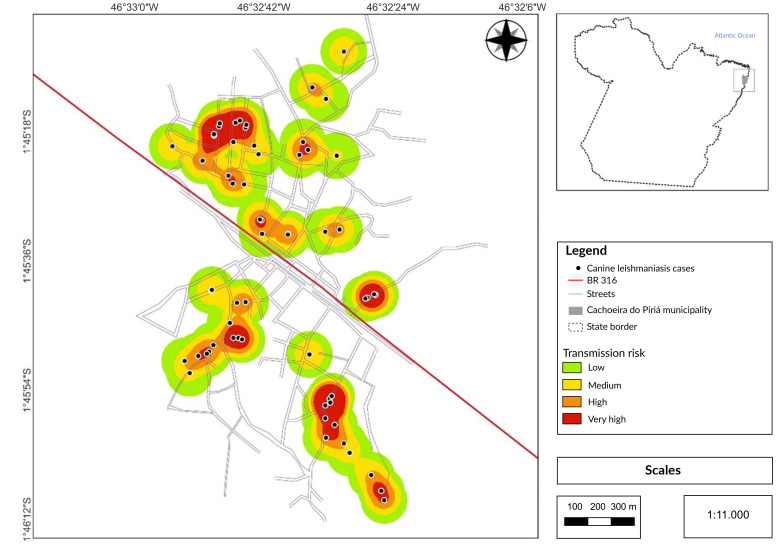
Spatial distribution highlighting clusters of canine leishmaniasis cases in the urban area. Cachoeira do Piriá, 2023

Cases of canine leishmaniasis were distributed across the entire urban area, with major clusters located in the northwestern neighborhoods (Piçarreira and Vitória) and southeastern neighborhoods (Cachoeira Velho), respectively situated above and below the BR-316 highway (Figure 2).

## Discussion

High prevalence of canine leishmaniasis in an urban area was observed during the dry season in the Cachoeira do Piriá Amazonian municipality, Pará state, where *Lutzomyia antunesi*, a sand fly species potentially acting as a vector of Leishmania pathogens responsible for cutaneous leishmaniasis in the Amazon, was also identified (5,6). The prevalence of canine leishmaniasis in the urban area based on PCR revealed a previously unknown scenario in the municipality that had only been endemic for tegumentary leishmaniasis. Given the absence of reported human cases of visceral leishmaniasis, Cachoeira do Piriá does not conduct canine surveillance.

Estimates of canine leishmaniasis prevalence are commonly based on serology, including rapid tests, and performed in visceral leishmaniasis endemic areas, while PCR is recommended as a confirmatory method, due to its high sensitivity and specificity (16). Reported seroprevalence rates of canine leishmaniasis in urban or peri-urban areas vary and are generally lower than those described in this study (17,18). The accuracy of serology for canine leishmaniasis has been widely questioned (6,19), particularly under conditions of low antibody levels, as observed in infections caused by Leishmania agents responsible for cutaneous leishmaniasis (20). For this reason, PCR was used as the diagnostic method.

The use of DNA extracted from the conjunctival secretion of dogs provided lower sensitivity for PCR compared to DNA extracted from blood, a contrast also demonstrated by other authors (21,22). In Brazil, conjunctival swabs provide variable sensitivity for PCR in different endemic areas, but generally they would provide results 12% more sensitive when compared to other biological samples, including blood (19). Variations in parasite load, the *Leishmania species* associated with canine leishmaniasis and environmental conditions of the study areas may influence PCR sensitivity, depending on the type of sample and the molecular target (23-24). When obtaining two minimally invasive sample types for testing is unfeasible, blood samples are recommended.

The choice of the *heat shock protein 70-234* gene sequence for PCR, in this stage of the study, was based on its potential to discriminate, in the future, between *Leishmania* species associated with canine enzootic disease by means of marker sequencing, given its good performance in distinguishing species, both in samples from dogs (2) and humans (25), as there are eight *Leishmania* pathogens reported in dogs in the Americas, five of which are endemic in Pará: *Leishmania infantum*, *Leishmania amazonensis*, *Leishmania braziliensis*, *Leishmania guyanensis* and *Leishmania naiffi* (2,16). Furthermore, dogs, which are reservoirs for *Leishmania infantum*, the etiological agent of visceral leishmaniasis, are also implicated as reservoirs for *Leishmania* species that cause cutaneous leishmaniasis (3,17).

In northeastern Pará, where Cachoeira do Piriá is located, canine leishmaniasis is caused by more than one Leishmania species (*Leishmania infantum*, *Leishmania braziliensis* and *Leishmania guyanensis*) (2). Despite the lack of clear evidence regarding the role of dogs in the epidemiology of human tegumentary leishmaniasis (16), the Brazilian Ministry of Health recommends differentiating *Leishmania* species in dogs in areas where human visceral and cutaneous leishmaniasis coexist (13).

Among the phlebotomine species captured during the Amazonian summer in the urban area of Cachoeira do Piriá, none were primary vectors of cutaneous leishmaniasis, nor was the most common visceral leishmaniasis vector, *Lutzomyia longipalpis*, which was also absent in a thorough survey conducted in 2022 (9).

The neighborhood with the highest positivity rate for canine leishmaniasis lacked sand flies presence, which suggests a lower risk of transmission during the Amazonian summer (25). Collections during both the dry and rainy seasons, using Shannon and CDC traps, would provide greater representation of the sand fly fauna (13), however the detection of *Lutzomyia antunesi*, previously reported in Cachoeira do Piriá (9), raises concern about the potential transmission of *Leishmania lindenbergi* or even *Leishmania naiffi* in urban areas. In the Amazon, *Lutzomyia antunesi* is considered a likely vector of these *Leishmania* species (5, 8-9).

Among sand flies of medical importance in the Americas, there are several endemic species in Pará (16). Their communities may be affected by environmental, climatic or anthropogenic changes related to the expansion of canine leishmaniasis in urban areas (26), as these conditions favor the adaptation of vectors to artificial ecotopes (27). In Cachoeira do Piriá, gold mining, including in urban environments, is noteworthy, significantly transforming landscapes and potentially impacting the transmission dynamics of *Leishmania* parasites to vertebrate hosts.

Regarding the spatial distribution of canine leishmaniasis cases in the urban area of Cachoeira do Piriá, clusters of positive dogs were observed in three neighborhoods, two in the northwest and one in the southeast of the municipality’s urban area. The spatial distribution of occurrences is critical for prioritizing intervention areas (28), and will guide planning for canine surveillance actions in Cachoeira do Piriá.

In conclusion, the prevalence of canine leishmaniasis in the urban area is high during the Amazonian summer in this municipality, where *Lutzomyia antunesi* may serve as a vector for *Leishmania* spp. Three of the five urban neighborhoods concentrated most canine infection cases and are priority areas for surveillance.

## Data Availability

The database and analysis codes used in this research are available at https://zenodo.org/records/13996845?preview=1.

## References

[1] Burza S, Croft SL, Boelaert M (2018). Leishmaniasis. Lancet [internet].

[2] Santos FJA, Nascimento LCS, Silva WB, Oliveira LP, Santos WS, Aguiar DCF (2020). First report of canine infection by Leishmania (Viannia) guyanensis in the Brazilian Amazon. Int J Environ Res Public Health.

[3] Kent A, Ramkalup P, Mans D, Schallig H (2013). Is the dog a possible reservoir for cutaneous leishmaniasis in Suriname?. J Trop Med.

[4] Gomes AC, Galati EAB (1989). Aspectos ecológicos da leishmaniose tegumentar americana: 7-Capacidade vetorial flebotomínea em ambiente florestal primário do Sistema da Serra do Mar, região do Vale do Ribeira, Estado de São Paulo, Brasil. Rev Saude Publica.

[5] Leão PO, Pereira  AM, de Paulo PFM, Carvalho LPC, Souza ABN, da Silva MS (2020). Vertical stratification of sand fly diversity in relation to natural infections of Leishmania sp. and blood-meal sources in Jamari National Forest, Rondônia State, Brazil. Parasit Vectors.

[6] Silveira FT, Ishikawa EAY, De Souza AAA, Lainson R (2002). An outbreak of cutaneous leishmaniasis among soldiers in Belém, Pará State, Brazil, caused by Leishmania (Viannia) lindenbergi n. sp. A new leishmanial parasite of man in the Amazon region. Parasite.

[7] Carvalho BM, Santos TV, Barata IR, Lima JAN, Silveira FT, Vale MM (2018). Entomological surveys of *Lutzomyia flaviscutellata* and other vectors of cutaneous leishmaniasis in municipalities with records of *Leishmania amazonensis* within the Bragança region of Pará State, Brazil. J Vector Ecol.

[8] Santos WS, Ortega DF, Alves VR, Garcez LM (2019). Flebotomíneos (Psychodidae: Phlebotominae) de área endêmica para leishmaniose cutânea e visceral no nordeste do estado do Pará, Brasil. Rev Pan Amaz Saude.

[9] Pinto CS, Alves VR, Santos WS, Garcez LM (2022). Phlebotominae (Diptera: Psychodidae) vetores de Leishmania spp., em área endêmica para a leishmaniose tegumentar no nordeste do Pará, Brasil. REAS.

[10] Cordeiro IMCC, Arbage MJC, Schwartz G (2017). Nordeste paraense: panorama geral e uso sustentável das florestas secundárias.

[11] Instituto Brasileiro de Geografia e Estatística - IBGE (2022). Cidades: 2022 [Internet].

[12] Brasil (2014). Manual de vigilância e controle da leishmaniose visceral.

[13] Brasil (2017). Manual de vigilância da leishmaniose tegumentar.

[14] Galati EAB (2023). Phlebotominae (Diptera, Psychodidae): classificação, morfologia, terminologia e identificação de adultos [Internet].

[15] Gonçalves R, Soares DC, Guimarães RJPS, Santos WS, Sousa GCR, Chagas AP (2016). Diversity and ecology of sand flies (Psychodidae: Phlebotominae): foci of cutaneous leishmaniasis in Amazon Region, Brazil. Rev Pan-Amaz Saude.

[16] Sevá ADP, Brandão APD, Godoy SN, Soares RM, Langoni H, Rodrigues BC, Gava MZE, Zanotto PFC, Jimenez-Villegas T, Hiramoto R, Ferreira F (2021). Investigation of canine visceral leishmaniasis in a non-endemic area in Brazil and the comparison of serological and molecular diagnostic tests. Rev Soc Bras Med Trop.

[17] Carvalho MR, Dias AFLR, Almeida ADBPF, Alves MR, Paes AS, Sousa VRF (2020). Canine visceral leishmaniasis: perception, prevalence, and spatial distribution in municipality of Nossa Senhora do Livramento, Mato Grosso, Brazil. Rev Bras Parasitol Vet.

[18] Ribeiro CR, Gonçalves CA, Cruz LM, Galera PD (2019). Prevalence of visceral canine leishmaniosis and co-infections in periurban region in the Federal District - Brazil. Cienc. anim. bras.

[19] Coura-Vital W, Ker HG, Roatt BM, Aguiar-Soares RD, Leal GG, Moreira Nd, Oliveira LA, de Menezes Machado EM, Morais MH, Corrêa-Oliveira R, Carneiro M, Reis AB (2014). Evaluation of change in canine diagnosis protocol adopted by the visceral leishmaniasis control program in Brazil and a new proposal for diagnosis. PLoS One.

[20] Baneth G, Yasur-Landau D, Gilad M, Nachum-Biala Y (2017). Canine leishmaniosis caused by *Leishmania major* and *Leishmania tropica*: comparative findings and serology. Parasit Vectors.

[21] Geisweid K, Weber K, Sauter-Louis C, Hartmann K (2013). Evaluation of a conjunctival swab polymerase chain reaction for the detection of *Leishmania infantum* in dogs in a non-endemic area. Vet J.

[22] Lombardo G, Grazia MG, Lupo T, Migliazzo A, Caprì A, Solano-Gallego L (2012). Detection of *Leishmania infantum* DNA by real-time PCR in canine oral and conjunctival swabs and comparison with other diagnostic techniques. Vet Parasitol.

[23] Courtenay O, Carson C, Calvo-Bado L, Garcez LM, Quinnell RJ (2014). Heterogeneities in *Leishmania infantum* infection: using skin parasite burdens to identify highly infectious dogs. PLoS Negl Trop Dis.

[24] Carson C, Quinnell RJ, Holden J, Garcez LM, Deborggraeve S, Courtenay O (2010). Comparison of Leishmania OligoC-TesT PCR with conventional and real-time PCR for Diagnosis of canine Leishmania infection. J Clin Microbiol.

[25] Almeida ANF, Nascimento LCSD, Sousa ESMM, Oliveira AJD, Sena MG, Resende BM, Chaves RCG, Garcez LM (2018). Surveillance of cutaneous leishmaniasis in clinical samples: distribution of *Leishmania guyanensis* in the state of Amapá, Brazil, 2018. Epidemiol Serv Saude.

[26] Borges MS, Niero LB, da Rosa LDS, Citadini-Zanette V, Elias GA, Amaral PA (2022). Factors associated with the expansion of leishmaniasis in urban areas: a systematic and bibliometric review (1959-2021). J Public Health Res.

[27] da Costa SM, Cordeiro JLP, Rangel EF (2018). Environmental suitability for *Lutzomyia* (Nyssomyia) whitmani (Diptera: Psychodidae: Phlebotominae) and the occurrence of American cutaneous leishmaniasis in Brazil. Parasit Vectors.

[28] Spindola CZ, Figueiredo FB, Arruda MM, Campos MP, Biffi LJ, Sebolt APR, Godinho NM, Chryssafidis AL, de Moura AB (2024). Canine visceral leishmaniasis: Seroprevalence and georeferencing in the state of Santa Catarina, Brazil. Vet Parasitol Reg Stud Reports.

