# Supplementary material for: Urban canine leishmaniasis in an Amazonian municipality: a cross-sectional study of prevalence, distribution and phlebotomine fauna during the dry season, Brazil, 2023
Source: Epidemiol Serv Saude. 2025 Jun 13;34:e20240130. doi: 10.1590/S2237-96222025v34e20240130.en (PMC12176442; doi:10.1590/S2237-96222025v34e20240130.en)
Supplement: Translation

# Translation: Leishmaniose canina urbana em município amazônico: estudo transversal de prevalência, distribuição e fauna flebotomínica durante a estação seca, Brasil, 2023

**DOI:** 10.1590/S2237-96222025v34e20240130.pt

**Authors:** Adriana Sousa Tapajós, Luciana Pinto Oliveira, Andréa Helena Martins Amaral, Clístenes Pamplona Catete, Walter Souza Santos, Lourdes Maria Garcez

**Affiliations:** 1 Secretaria de Estado de Saúde Pública do Pará, Diretoria de Endemias, Belém, PA, Brasil; 2 Instituto Evandro Chagas, Laboratório de Epidemiologia das Leishmanioses, Ananindeua, PA, Brasil; 3 Universidade do Estado do Pará, Centro de Ciências Biológicas e da Saúde, Belém, PA, Brasil

**Keywords:** Leishmaniose canina, Reação em Cadeia da Polimerase, Localização Geográfica de Risco, Psychodidae, Estudos transversais

## Abstract

**Objetivo:**

Descrever prevalência e distribuição da leishmaniose canina e a fauna flebotomínica nos cinco bairros urbanos do município amazônico Cachoeira do Piriá, Pará, Brasil, durante a estação seca.

**Métodos:**

Cães foram amostrados por cinco horas em cada bairro, com espécimes de sangue e swab conjuntival (de 13 a 15/6/2023). Amostras foram testadas pela reação em cadeia da polimerase baseada no alvo gênico *heat shock protein 70*-234. Áreas de risco (kernel) e fauna flebotomínica foram investigadas (de 1o a 7/10/2023).

**Resultados:**

Incluíram-se 93/864 (11%) animais. A prevalência de leishmaniose canina foi 65% (60/93). Taxas de positividade variaram entre bairros (p-valor 0,001), com aglomerados principais a Noroeste e Sudeste da área urbana, abrangendo três bairros. *Lutzomyia antunesi* (2) e *Lutzomyia evandroi* (11) foram encontrados em quatro bairros.

**Conclusão:**

Alta prevalência de leishmaniose canina e um potencial vetor vetor flebotomíneo (*Lutzomyia antunesi*) foram descritos durante o verão amazônico na área urbana de Cachoeira do Piriá, onde três bairros são prioritários para a vigilância.

**Aspectos éticos**

A presente pesquisa respeitou os princípios éticos, obtendo os seguintes dados de aprovação: :

Comitê de ética em pesquisa: Instituto Evandro Chagas

Número do parecer: 04/2023

Data de aprovação: 14/4/2023

Certificado de apresentação de apreciação ética: 04/2023

Registro doconsentimento livre e esclarecido: Não há participação de seres humanos na pesquisa. Todos os tutores dos cães autorizaram por escrito a coleta de amostras.

## Introdução

O cão doméstico é hospedeiro reservatório da *Leishmania infantum*, agente etiológico da leishmaniose visceral (1), e incriminado também como reservatório de espécies causadoras da leishmaniose tegumentar (2-3).

A capacidade vetorial dos insetos flebótomos depende da densidade da espécie, habilidade em colonizar ambientes modificados e competência de se alimentar do sangue humano (4). A espécie *Lutzomyia antunesi*, encontrada naturalmente infectada com *Leishmania naiffi* em Rondônia (5) e Leishmania lindenbergi no Pará (6), e frequentemente reportada no nordeste do estado do Pará (7-9), seria um potencial vetor de Leishmania. 

Este estudo descreve a prevalência e distribuição da leishmaniose canina e a fauna flebotomínica em área urbana de Cachoeira do Piriá, município do nordeste paraense, durante o verão amazônico.

## Métodos

### Delineamento

Estudo descritivo transversal em área urbana do município Cachoeira do Piriá, nordeste do estado do Pará, Amazônia, Brasil.

### Contexto

O nordeste do Pará equivale à mais antiga fronteira de colonização do estado (10), onde Cachoeira do Piriá sofre frequentes alterações nas paisagens, inclusive decorrentes da extração do ouro em área urbana. De 2013 a 2022, notificaram-se 324 casos de leishmaniose tegumentar no município, que possui 19.630 habitantes, 2.419 km^2^ (11) e apenas cinco bairros urbanos. As equipes de vigilância locais não atuam na vigilância da leishmaniose canina.

### Amostragem de cães urbanos

A coleta de sangue (2-3mL) e swab conjuntival dos cães ocorreu no tempo fixo de 5h/unidade amostral (bairro), durante busca ativa orientada pelo censo urbano canino de 2022 (864 cães). Sangue em ácido etilenodiamina tetra-acético e swab conjuntival sem conservante foram acondicionados, transportados (4^o^-8^o^ C/3h) e preservados (-20^o^ C) até a extração do ácido desoxirribonucleico (DNA). 

### Variáveis e estatísticas

Variáveis: a) prevalência, distribuição e densidade de casos de leishmaniose canina; b) frequência e distribuição de espécies flebotomínicas; c) sensibilidade da reação em cadeia da polimerase (PCR) baseada no alvo *heat shock protein 70*-234 (2), com DNA extraído de sangue e swab conjuntival para diagnosticar leishmaniose canina.

Comparou-se a sensibilidade da PCR com o DNA extraído do sangue e do swab conjuntival (teste de Fisher) e as proporções de cães positivos entre os cinco bairros urbanos (qui-quadrado de independência) de Cachoeira do Piriá (α: 0,05; BioEstat, versão 5.0).

### Diagnóstico de leishmaniose canina

Após extração do DNA (*Wizard Genomic DNA Purification* - Promega), volume final de 50mL, as condições da PCR foram: Mix 50mL (Taq DNA polimerase 0,03U/µL; MgCl2 1,5mM; tampão KCl *buffer* Invitrogen; dNTPs 0,25mM; iniciadores F- 5’ GGA CGA GAT CGA GCG CAT GGT 3’ e R- 5’ TCC TTC GAC GCC TCC TGG TTG 3’, 0,2pM cada; DNA 3,0µL); desnaturação, anelamento e extensão (1x 94° C/5’; 32x 94° C/0,5’, 61^o^ C/1’ e 72^o^ C/1’); e extensão final (72° C/10’). A amplificação do alvo (*heat shock protein 70*-234), visualizada em gel de agarose, confirmou presença de DNA de Leishmania na amostra (2).

### Coleta e identificação de flebótomos

De 1^o^ a 7/10/2023, instalaram-se 14 armadilhas luminosas *Center on Disease Control* (conhecidas como CDC), em peri e intradomicílio (18h-6h), por três noites consecutivas, nos cinco bairros de Cachoeira do Piriá, em sete pontos de captura: Vitória (1); Piçarreira (2); Centro (1); São José (2) e Cachoeira Velho (1). O esforço total de captura entomológica foi de 180 horas. Flebótomos foram preservados (em álcool 70%) clarificados, diafanizados, montados entre lâmina e lamínula com Berlese e identificados morfologicamente (12-14).

## Resultados

Foram incluídos 93/846 (11%) cães urbanos, machos e fêmeas, com idade igual ou superior a 6 meses, sintomáticos ou não para leishmaniose.

A prevalência de leishmaniose canina foi de 65% (60/93) no ambiente urbano, conforme resultados da PCR (Tabela 1). Proporções de positividade por bairro (Figura 1) variaram de 35% a 94% (ꭓ^2^: 19,036; 4GL; p-valor 0,001). 

**Tabela 1:** Reação em cadeia da polimerase (PCR), baseada na sequência gênica *heat shock protein 70*-234, para o diagnóstico da leishmaniose canina e da fauna flebotomínica da estação seca, em cinco bairros urbanos. Cachoeira do Piriá, 2023

Bairros urbanos	PCR para leishmaniose canina		Total n (%)	Espécies flebotomínicas no peridomicílio	Sexo		Total n (%)
	Positivos	Negativos			Macho	Fêmea	
Piçarreira	15	1	16 (17,0)	-	0	0	0
Vitória	17	3	20 (22,0)	*Evandromyia evandroi*	1	3	4 (31,0)
Cachoeira Velho	15	9	24 (26,0)	*Evandromyia evandroi*	1	5	6 (46,0)
Centro	7	9	16 (17,0)	*Nyssomyia antunesi*	0	1	1 (8,0)
São José	6	11	17 (18,0)	*Nyssomyia antunesi*	0	1	1 (8,0)
				*Evandromyia evandroi*	1	0	1 (8,0)
Total	60	33	93 (100)		3	10	13 (100)

**Figura 1:** Taxas de resultados positivos e negativos da reação em cadeia da polimerase para leishmaniose canina, baseada na sequência gênica *heat shock protein 70*-234, em cinco bairros urbanos. Cachoeira do Piriá, 2023 (p-valor 0,001)

Houve diferença significante na sensibilidade do teste em função da amostra (p-valor 0,001) de DNA extraído do sangue (53/93; 57%); ou de swab conjuntival (32/93; 34%). 

Flebótomos foram encontrados no peridomicílio em quatro bairros, exceto Piçarreira. Duas espécies foram representadas na amostra (Tabela 1).

Casos de leishmaniose canina distribuíram-se em toda a área urbana, com principais aglomerados nos bairros a Noroeste (Piçarreira e Vitória) e Sudeste (Cachoeira Velho), respectivamente acima e abaixo da Rodovia BR-316 (Figura 2). 

**Figura 2:** Distribuição espacial dos aglomerados de casos de leishmaniose canina urbana. Cachoeira do Piriá, 2023

## Discussão

Constatou-se alta prevalência de leishmaniose canina urbana durante a estação seca no município amazônico Cachoeira do Piriá, estado do Pará, onde também se identificou *Lutzomyia antunesi*, espécie flebotomínea potencialmente vetora de patógenos Leishmania causadores de leishmaniose tegumentar na Amazônia (5,6). A prevalência da leishmaniose canina urbana baseada na PCR revelou um cenário antes desconhecido no município, endêmico apenas para leishmaniose tegumentar. Por não registrar casos humanos de leishmaniose visceral, Cachoeira do Piriá não atua na vigilância canina. 

Estimativas de prevalência da leishmaniose canina são comumente baseadas na sorologia, incluindo os testes rápidos, e realizadas em áreas endêmicas para leishmaniose visceral, enquanto a PCR é indicada como confirmatória, pela sua alta sensibilidade e especificidade (16). Soroprevalências de leishmaniose canina relatadas para áreas urbanas ou periurbanas são variáveis, e geralmente mais baixas do que a descrita neste estudo (17,18). A sorologia para leishmaniose canina é muito questionada quanto a sua acurácia (6,19) e não seria efetiva na vigência de baixos níveis de anticorpos, como nas infecções por agentes etiológicos da leishmaniose tegumentar (20), motivo pelo qual a PCR foi o método utilizado.

O uso do DNA extraído da secreção conjuntival dos cães proporcionou baixa sensibilidade à PCR, comparado ao DNA extraído do sangue, um contraste também demonstrado por outros autores (21,22). No Brasil, o swab conjuntival proporciona sensibilidade variável à PCR em diferentes áreas endêmicas, mas em geral proveria resultados 12% mais sensíveis, se comparado a outras amostras biológicas, incluindo-se o sangue (19). Variações na carga parasitária, espécie de *Leishmania* associada à leishmaniose canina e condições ambientais das áreas de estudo poderiam influenciar a sensibilidade da PCR, em função do tipo de amostra e do alvo molecular (23-24). Na inviabilidade de se obterem dois tipos de amostra minimamente invasivas para testagem, recomenda-se a obtenção de sangue.

A opção pela sequência gênica *heat shock protein 70*-234 para PCR, nesta fase do estudo, decorre da pretensão de discriminar futuramente espécies de Leishmania associadas à enzootia canina por meio de sequenciamento do marcador, dado seu bom desempenho na distinção de espécies, tanto em amostras de cães (2) como de humanos (25), pois são oito os patógenos de *Leishmania* reportados em cães nas Américas, cinco deles endêmicos no Pará: *Leishmania infantum*, *Leishmania amazonensis*, *Leishmania braziliensis*, *Leishmania guyanensis* e *Leishmania naiffi* (2,16). Ademais o cão, reservatório de *Leishmania infantum*, agente etiológico da leishmaniose visceral, é também incriminado como reservatório de espécies de Leishmania causadoras da leishmaniose tegumentar (3,17).

No Nordeste do Pará, região onde se situa Cachoeira do Piriá, a leishmaniose canina é causada por mais de uma espécie de Leishmania (*Leishmania infantum*, *Leishmania braziliensis* e *Leishmania guyanensis*) (2). Mesmo sem evidências claras da importância do cão na epidemiologia da leishmaniose tegumentar humana (16), o Ministério da Saúde do Brasil recomenda discriminar espécies de Leishmania em cães nas áreas onde coexistam leishmaniose visceral e tegumentar humanas (13).

Entre as espécies flebotomíneas capturadas durante o verão amazônico, na área urbana de Cachoeira do Piriá, não havia os principais vetores da leishmaniose tegumentar e tampouco o mais comum vetor da leishmaniose visceral, *Lutzomyia longipalpis*, também ausente durante minucioso levantamento no município, em 2022 (9). 

O bairro com mais alta taxa de positividade para leishmaniose canina foi negativo para flebótomos, o que sugere menor risco de transmissão no verão amazônico (25). Coletas nas estações seca e chuvosa, com armadilhas Shannon e CDC, proporcionariam maior representatividade da fauna flebotomínica (13), mas o achado de *Lutzomyia antunesi*, previamente reportado em Cachoeira do Piriá (9), alerta para a possível transmissão de *Leishmania lindenbergi* ou mesmo *Leishmania naiffi* em área urbana, posto que, na Amazônia, *Lutzomyia antunesi* é provável vetor dessas espécies de Leishmania (5,8-9).

Entre os flebótomos de importância médica nas Américas, há diversas espécies endêmicas no Pará (16), cujas comunidades podem ser afetadas por alterações ambientais, climáticas ou antrópicas, relacionadas à expansão da leishmaniose canina urbana (26), pois favorecem a adaptação de vetores a ecótopos artificiais (27). Em Cachoeira do Piriá, a mineração de ouro, inclusive em ambientes urbanos, é notável, transformando significativamente as paisagens e potencialmente impactando a dinâmica de transmissão de parasitas Leishmania para hospedeiros vertebrados. Quanto à distribuição espacial dos casos de leishmaniose canina em área urbana de Cachoeira do Piriá, observaram-se aglomerados de cães positivos em três bairros, dois a Noroeste e um a Sudeste da área urbana do município. A distribuição espacial das ocorrências é crítica para a seleção de áreas prioritárias (28), e servirá para orientar o planejamento de ações de vigilância canina em Cachoeira do Piriá. 

Conclui-se que é alta a prevalência de leishmaniose canina urbana durante o verão amazônico no município, onde *Lutzomyia antunesi* poderia atuar como vetor de *Leishmania* spp. Dos cinco bairros urbanos, três concentraram a maioria dos casos de infecção canina e são áreas prioritárias para a vigilância.

## Data Availability

O banco de dados e os códigos de análise utilizados na pesquisa estão disponíveis em https://zenodo.org/records/13996845?preview=1.
